# 1α,25(OH)_2_ Vitamin D3 Modulates Avian T Lymphocyte Functions without Inducing CTL Unresponsiveness

**DOI:** 10.1371/journal.pone.0150134

**Published:** 2016-02-24

**Authors:** Nitish Boodhoo, Shayan Sharif, Shahriar Behboudi

**Affiliations:** 1 The Pirbright Institute, Ash Road, Woking, United Kingdom; 2 Department of Pathobiology, Ontario Veterinary College, University of Guelph, Guelph, Ontario, Canada; University of Sydney, AUSTRALIA

## Abstract

1,25-Dihydroxyvitamin D3 (Vitamin D) is a naturally synthesized fat soluble vitamin shown to have immunomodulatory, anti-inflammatory and cancer prevention properties in human and murine models. Here, we studied the effects of Vitamin D on the functional abilities of avian T lymphocytes using chicken Interferon (IFN)-γ ELISPOT assay, BrdU proliferation assay, Annexin V apoptosis assay and PhosFlow for detecting phosphorylated signalling molecules. The results demonstrate that Vitamin D significantly inhibited the abilities of T lymphocytes to produce IFN-γ and proliferate *in vitro* (P≤0.05), but retained their ability to undergo degranulation, which is a maker for cytotoxicity of these cells. Similarly, Vitamin D did not inhibit Extracellular signal-Regulated Kinase (ERK) 1/2 phosphorylation, a key mediator in T cell signalling, in the stimulated T lymphocytes population, while reduced ERK1/2 phosphorylation levels in the unstimulated cells. Our data provide evidence that Vitamin D has immuno-modulatory properties on chicken T lymphocytes without inducing unresponsiveness and by limiting immuno-pathology can promote protective immunity against infectious diseases of poultry.

## Introduction

Vitamin D is a naturally synthesized lipid soluble vitamin and has a broad range of physiological properties, including profound effects upon immune system [1–3]. Ultraviolet B (UV-B) irradiation of epidermal cells constitutes the primary step for photolytic conversion of 7-dehydrocholesterol to Vitamin D. Modern poultry farming practices have led to an increase in density housing with minimal ultraviolet light B (UV-B) exposure. Thus, eggs produced from indoors housed chickens have a significantly lower (3.8 μg 1α,25(OH)_2_D3 /100g of dry matter) egg yolk 1α,25(OH)_2_D3 content compared to those housed outdoors (14.3 μg 1α,25(OH)_2_D3 /100g of dry matter). The 25-hydroxyvitamin D (25(OH)D3) content of egg yolk was also influenced by sunlight exposure, although less pronounced than the Vitamin D3 content [4]. Alternatively, Vitamin D3 can be acquired in the diet or as supplements. Vitamin D3 is subsequently hydroxylated by hepatic mitochondrial cytochrome P450 (CYP27A1) into 25(OH)D3. Finally, 25(OH)D3 is hydroxylated by renal mitochondrial cytochrome P450 (CYP27B1) into 1α, 25-dyhydroxyvitamin D (1α,25(OH)_2_D3). The latter is biologically relevant and active form with endocrine actions. Macrophages have been shown to express both CYP27A1 and CYP27B1 [1, 5] enzymes required to produce 1α,25(OH)_2_D3, whereas T-cells can only perform the final metabolic step [6, 7]. Therefore, immune system cells may be able to use Vitamin D in an autocrine and paracrine manner.

The immune modulatory functions of 1α,25(OH)_2_D3 have been linked to genomic effects mediated by Vitamin D Receptor (VDR), a member of the nuclear hormone superfamily found in most immune cells [3, 8] such as macrophages [9], dendritic cells [10], B-cells [8, 9, 11] and T cells [8, 9]. Mice VDR^-/-^ models have been used to demonstrate this interdependent relationship [12–14]. Genetic polymorphism in the VDR gene has been associated with susceptibility to several viral infections in human; Dengue Virus (DENV; rs2228570) [15], Hepatitis B Virus (HBV; FokI C>T) [16], Respiratory Syncytial Virus (RSV; rs10735810) [17] and even in chickens; Marek’s Disease Virus (MDV; S1P4) [18]. In humans, Vitamin D has been shown to be effective in the prevention and control of viral diseases such as Human Immunodeficiency Virus (HIV) [19, 20] and RSV [21]. Studies into the mechanistic effects have demonstrated that Vitamin D regulates immune system cells functional abilities in an attempt to maintain immune homeostasis.

In the context of innate immunity, Vitamin D may influence the type and magnitude of antigen presenting cell responses and their retrospective ability to modulate T lymphocyte function. It has been recently demonstrated that chicken macrophages exposed to 25(OH)D3 have a 5-fold increase in nitric oxide production [22]. Stimulating nitric oxide production enhances phagocytic activity of macrophages and induces cytostatic or cytotoxic action against viruses, bacteria, fungi and tumour cells [23]. In addition, low dose Vitamin D treatment may restore human macrophage proliferative ability [24], and increase antimicrobial peptide production such as cathelicidin and β-defensin in response to stimuli [25]. Vitamin D may perturb dendritic cells responsiveness to microbial stimuli, thus impeding maturation [26]. Human dendritic cells and macrophages have been shown to produce less interleukin (IL)-12 when treated with high dose Vitamin D [27]. This could reduce their functional capacity as antigen presenting cells (APC) required for initiation of Th1 type T cell responses.

In the context of adaptive immunity, defence against intracellular pathogens is mediated in part by CD4^+^ and CD8^+^ T lymphocytes. Vitamin D alters naive and effector T-cell activation, and their cytokine secretion patterns [28]. This pleiotropic lipid soluble vitamin may be important for potentiating induction of naive T-cells via an alternative mitogen-activated protein kinase (MAPK) pathway [29]. The latter is involved in establishing intracellular PLC-γ1 protein which plays a central role in classical T-cell receptor (TCR) signaling pathway. However, human PBMC’s stimulated with a T cell specific mitogen (PHA; phytohemagglutinin) in the presence of Vitamin D was observed to inhibit cellular proliferation which has been associated with a decrease in IL-2 production [30]. Additionally, Vitamin D has been shown to inhibit IL-17 production [31] and increase IL-4, IL-5, IL-10 and IL-13 cytokines expression [32–34]. T cell trafficking and homing to specific tissues may be influenced by Vitamin D [3]. Vitamin D positively redirected T-cell specific translocation to epidermal keratinocytes by stimulating expression of chemokine receptor 10 (CCR10), which recognizes the chemokine CCL27 [35]. This will influence the retention of T cells with specific phenotypes and effector function within the skin [36].

Vitamin D has profound effects on the function of human and murine T cells but there is an information deficit regarding its effects on the functional abilities of avian immune system cells, especially chicken T cells and their responses to pathogens. Here, we demonstrate that Vitamin D inhibits IFN-γ production and T cell proliferation without inducing program cell death. In contrast to its inhibitory effects on the inflammatory functions of T lymphocytes, Vitamin D did not inhibit CD107a expression, a degranulation and cytotoxicity marker, and is shown to be crucial for the control of intracellular pathogens. Moreover, Vitamin D did not inhibit Extracellular signal-Regulated Kinase (ERK) 1/2 signalling pathway, which is regulated in T lymphocytes by T cell receptor (TCR)-CD3 complex interacting with peptide epitopes presented by antigen presenting cells.

## Materials and Methods

### Experimental Animals

Inbred mixed sex Rhode Island Red chickens were reared pathogen free in filtered-air positive pressure rooms on floor pens with wood shaving at The Pirbright Institute. Birds were group housed, and had ad libitum access to water and commercial feed. The work was performed according to home office guidelines. Tissue samples were taken from animals after humane killing under schedule 1. Three week old birds were killed by cervical dislocation, and spleens were removed and collected aseptically.

### Spleen mononuclear cell preparation

Whole spleens were kept on ice in PBS as soon as they were removed from chickens. After being rinsed in PBS, spleens were placed onto 40-μm BD cell strainers (BD Biosciences, UK), and crushed through using the flat end of a syringe plunger. Splenocytes cell suspension were prepared by layering (2:1) onto LymphoprepTM (Axis-shield PoC AS, Norway) density-gradient centrifugation, and centrifuged at 2100 rpm for 20 min to allow the separation of mononuclear cells. Mononuclear cells were subsequently aspirated from the interface, and washed at 1500 rpm for 5 min in RPMI 1640 with penicillin (10 U/ml), and streptomycin (10 μg/ml). Mononuclear cells were suspended in complete RPMI cell culture medium; RPMI 1640 medium containing 10% foetal bovine serum (Sigma, UK), penicillin (10 U/ml), and streptomycin (10 μg/ml). Cell number and viability were calculated using a haemocytometer, and trypan blue exclusion method. Mononuclear cells were suspended in complete RPMI cell culture medium at a density of 5 × 10^6^ cells/ml and kept on ice.

1α, 25-Dihydroxyvitamin D3 (Sigma, UK) was suspended in Dimethyl Sulfoxide (DMSO) (Sigma, UK) at 2.5x10^-4^M. Mononuclear cells were stimulated in the presence of 1α,25-Dihydroxyvitamin D3 (10^-7^M and 10^-8^M), or DMSO (vehicle-control) in Sterilin™ 7 mL polystyrene bijou containers (Thermo Scientific, UK) and incubated (4 hours; 41°C, 5% CO_2_). Cells were harvested, washed and centrifuged at 1500 rpm for 5 min, and suspended in complete RPMI cell culture medium for use in subsequent assays.

### Bromodeoxyuridine (BrdU) incorporation and proliferation Assay

Detection and quantification of mononuclear cell proliferation was measured by BrdU integration (Roche, UK) into DNA. In brief, mononuclear cells were seeded at 4.0 x10^4^ cells per well into 96-well microtiter plates and stimulated with medium alone or in the presence of Concanavalin A (ConA; 1–20μg/ml). Cells were incubated (41°C, 5% CO_2_) for 72 hours with an 18 hour pulse of 10 μM BrdU. BrdU integration was detected by labelling with 100μl anti-BrdU-POD monoclonal antibody and incubating at room temperature (RT) for 90 min. A Tetramethyl-Benzidine (TMD) substrate solution was used to develop the colour at RT for 15 min in the dark. The reaction product was inactivated with 1M sulphuric acid. Immune complexes were detected by reading absorbance at 450 nm (OD_450_).

### Chicken IFN-γ ELISPOT Assay

IFN-γ production was detected using an IFN-γ Chicken antibody pair kit (life technologies). In brief, MAIPS4510 MultiScreenTM-IP 96 well plates (Millipore, UK) were coated overnight at 4°C with 2μg/ml mouse anti-ChIFN-γ in PBS. Plates were washed twice with blocking buffer (RPMI 1640 plus 2% FCS) and incubated (1 hour; 37°C, 5% CO_2_) with blocking buffer. Mononuclear cells were seeded in triplicates at 5.0 x10^5^ cells per well, stimulated with medium alone or in the presence of PMA (50 ng/ml) plus Ionomycin (Ion; 1 μg/ml); (Sigma, UK) and incubated (41°C, 5% CO_2_) overnight. Plates were washed twice with SQ water followed by three time wash with washing buffer (PBS + 0.1% Tween 20). 1μg/ml of anti-chicken IFN- γ biotinylated antibody in assay buffer (PBS + 0.1% Tween 20 + 1.0% BSA) was added to the plate and incubated for 1 hour in the dark at RT. Plates were washed with washing buffer and incubated for a further 1 hour with Streptavidin-HRP (1/1250) in assay buffer. A final wash with washing buffer was performed and a 3-Amino-9-ethylcarbazole (AEC) substrate solution (BD Biosciences, UK) was used to develop the colour at RT in the dark. After 20 min, the reaction was inactivated by washing in distilled water, air dried overnight and spots forming units (SFU) were counted using an automated ELISPOT reader.

### Flow Cytometry/Phosflow

Unconjugated CD107a (LEP) and isotype control (CC63) antibodies were conjugated using the Alexa Fluor 647 labelling kit (Life Technologies) according to manufacturer’s recommendation prior to use. The degranulation marker, CD107a, of mononuclear cells were assessed: mononuclear cells were seeded in triplicates at 5.0 x10^5^ cells per well and stimulated with medium alone or in the presence of PMA (50 ng/ml) plus Ion (1 μg/ml) with the following antibodies; LEP (CD107a-Alexa Fluor 647) or isotype control (cc63-Alexa Fluor 647) and incubated (4 hours; 41°C, 5% CO_2_). Following a wash in PBS, cells were labelled with CD3-FITC (Southern Biotech) for 15 min at 4°C and dead cells were excluded using 7-AAD-PE staining. Cells were acquired on a FACS Calibur flow cytometer and data processed by FloJo software.

To determine the effects of Vitamin D on cell apoptosis, mononuclear cells were stained with Annexin V-APC (BDTM Pharmigen, UK) and 7-AAD-PE (BDTM Pharmigen, UK). Apoptotic and dead cells were acquired with a FACS Calibur flow cytometer and data were analysed using FloJo software.

Mononuclear cells were seeded in triplicates at 5.0 x10^5^ cells per well and incubated (5 and 15 min; 41°C, 5% CO_2_) with medium alone or in the presence of PMA (50 ng/ml). Cells were fixed for 30 minutes using the fixation/permeabilization kit (eBioscience). Cells were incubated in Perm buffer for 10 min at 4°C, followed by staining with ERK 1/2-PE (T202/Y204) (BDTM Pharmigen, UK), antibody for an additional 30 min at 4°C. Cells were washed twice in Perm buffer, and resuspended in FACS buffer. Cells were acquired on a FACS Calibur flow cytometer and data processed by FlowJo software.

### Statistical Analysis

All data are presented as mean + SD. ELISpot SFU data were adjusted to 1.0 x10^6^ cells. Quantification was performed using Graph Pad Prism 6 for windows. The data were analysed by Wilcoxon and Mann Whitney non-parametric to test significance as appropriate. Results were considered statistically significant at *P* < 0.05 (*).

## Results

### Vitamin D inhibits T-cell proliferation and IFN-γ production

**Fig 1:**
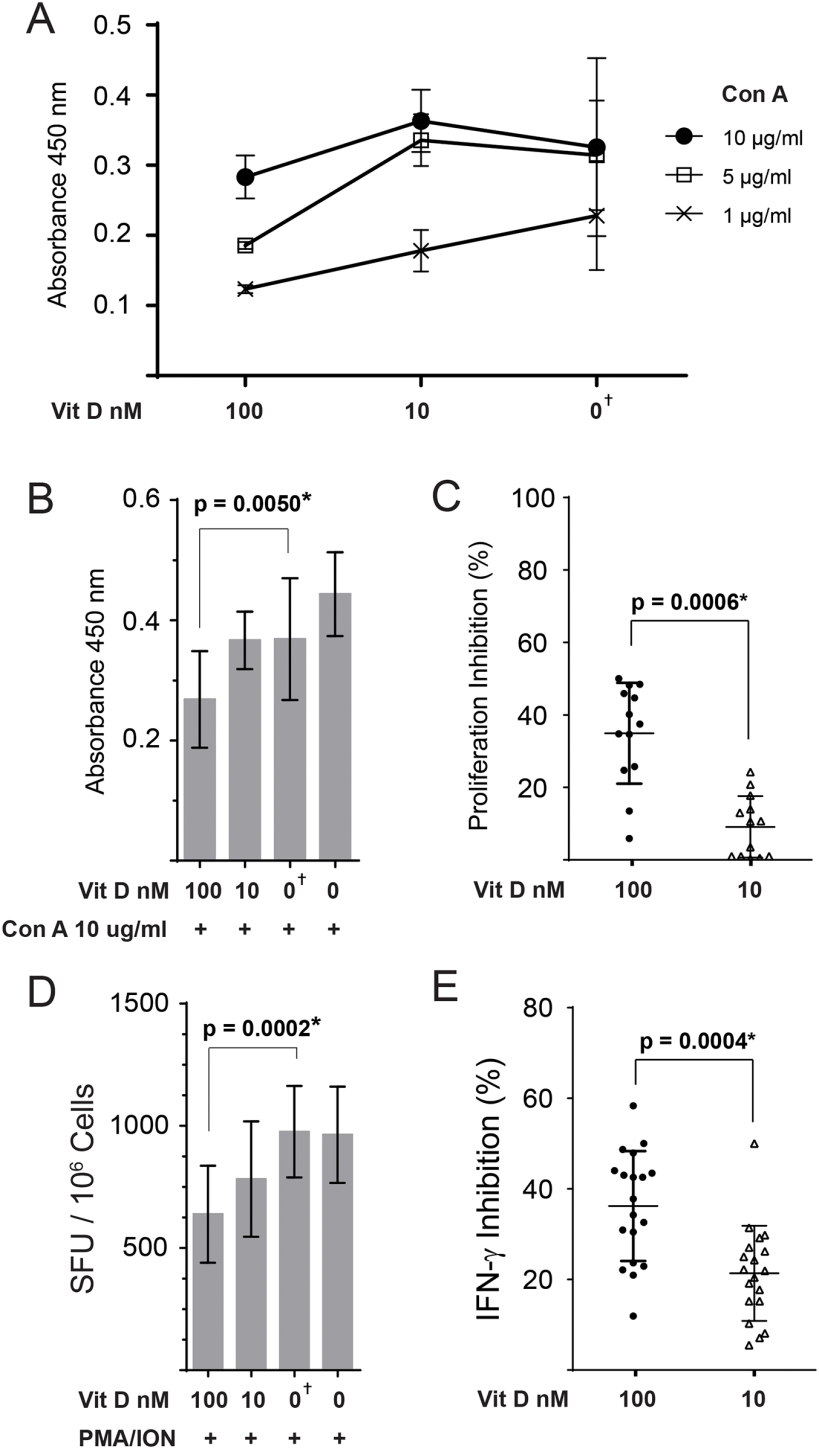
Vitamin D inhibits chicken T-cell proliferation and IFN-γ production *ex vivo*. Splenocytes were pre-treated for 4hrs with Vitamin D (100 and 10 nM) or vehicle only (DMSO). (**A**) BrdU incorporation measured by ELISA assay to quantify T cell proliferation after stimulation with different concentrations of Concanavalin A. The results are presented as absorbance at OD_450_ from three independent experiments with 3–4 biological replicates in each experiment. (B, C) The combined results from proliferation from 6 independent experiments for absorbance at OD_450_ (B) and the percentages of inhibition by Vitamin D are shown. (**D**) The frequency of IFN-γ producing mononuclear cells stimulated with a T-cell stimulation cocktail of PMA and Ion was detected using a chicken IFN-γ ELISPOT assay. The results are presented as spots forming unit (SFU) per 1.0 x 10^6^ cells from five independent experiments with 3–5 biological replicates in each experiment (19 replicates in total). (**E**) Represents the percentage of inhibition for IFN-γ production by mononuclear cells after Vitamin D_3_ pre-treatment from five independent experiments. Each dot represent a biological replicate. Non-parametric Wilcoxon tests (Mann-Whitney) was used to assess normal distribution and test significance. The results are shown as mean ± SD. † (symbol) represents cells treated with Vehicle only (DMSO). * indicates a statistically significant difference (*P* < 0.05).

Mononuclear chicken splenocytes were cultured in complete RPMI medium containing high (100 nM) or low (10 nM) concentration of Vitamin D for 4 hrs and their ability to proliferate *ex vivo* was examined using a BrdU-based proliferation assay. Some cells were cultured in vehicle only (DMSO) or medium only and were used as the control groups. The ability of Vitamin D pre-treated chicken T cell to proliferate *in vitro* in response to different concentrations of Con A (1, 5, 10 or 20 μg/ml) was analysed in three independent experiments. The highest concentration of Con A (20 μg/ml) induced high levels of cell death in two independent experiments (data not shown), while the lowest Con A concentration (1 μg/ml) was suboptimal for T cell activation (Fig 1A). The results demonstrate that high level of Vitamin D (100 nM) significantly inhibited T cell proliferation, regardless of the concentration of Con A (1–10 μg/ml) used for the stimulation (Fig 1A). In contrast, low Vitamin D (10 nM) did inhibit T cell proliferation irrespective of Con A concentrations used for stimulation. The optimal concentration of Con A to induce highest levels of T cell proliferation was 10 μg/ml in both control cells and Vitamin D treated cells (Fig 1A). Therefore, we used this concentration of the stimuli in further experiments. Three additional independent experiments were performed to confirm the inhibitory effects of Vitamin D on T cell proliferation. The combined data for absorbance (Fig 1B) and percentage inhibition (Fig 1C) from 6 independent experiments are shown. The results demonstrate that the cells treated with high concentration (100 nM) Vitamin D, but not low concentration (10 nM) Vitamin D, had lower proliferative ability in response to Con A (10 μg/ml) stimulation compared to the cells treated with vehicle only (*P* = 0.005; Fig 1B) or medium only.

Having demonstrated that Vitamin D (100 nM) significantly inhibits T cell proliferation *ex vivo*, we analysed the influence of this vitamin on chicken T cells ability to produce IFN-γ using a chicken IFN-γ ELISpot assay. Vitamin D-treated (100nM or 10 nM) or vehicle-treated chicken splenocytes were stimulated with a cocktail of PMA (50 ng/ml) and Ion (1μg/ml) for 18hrs. The data from five independent experiments with 3–5 biological replicates in each experiment were combined (Fig 1D). The results demonstrate that Vitamin D (100 nM) significantly inhibited IFN-γ production (*P* = 0.0002; Fig 1D) compared to the cells pre-treated with vehicle only. Low levels of Vitamin D also significantly reduced IFN-γ production compared to the vehicle only (*P* = 0.0073). No significant difference was observed between vehicle treated and untreated control cells, indicating that vehicle does not influence the ability of chicken T cells to produce IFN-γ.

The combined inhibition percentages for IFN-γ production are shown in Fig 1E. The results demonstrate that cells treated with 100 nM Vitamin D have significantly lower ability to produce IFN-γ compared to the cells treated with 10 nM Vitamin D (*P* = 0.0004).

### The inhibitory effects of Vitamin D on T cells is not due to cell death or apoptosis

**Fig 2:**
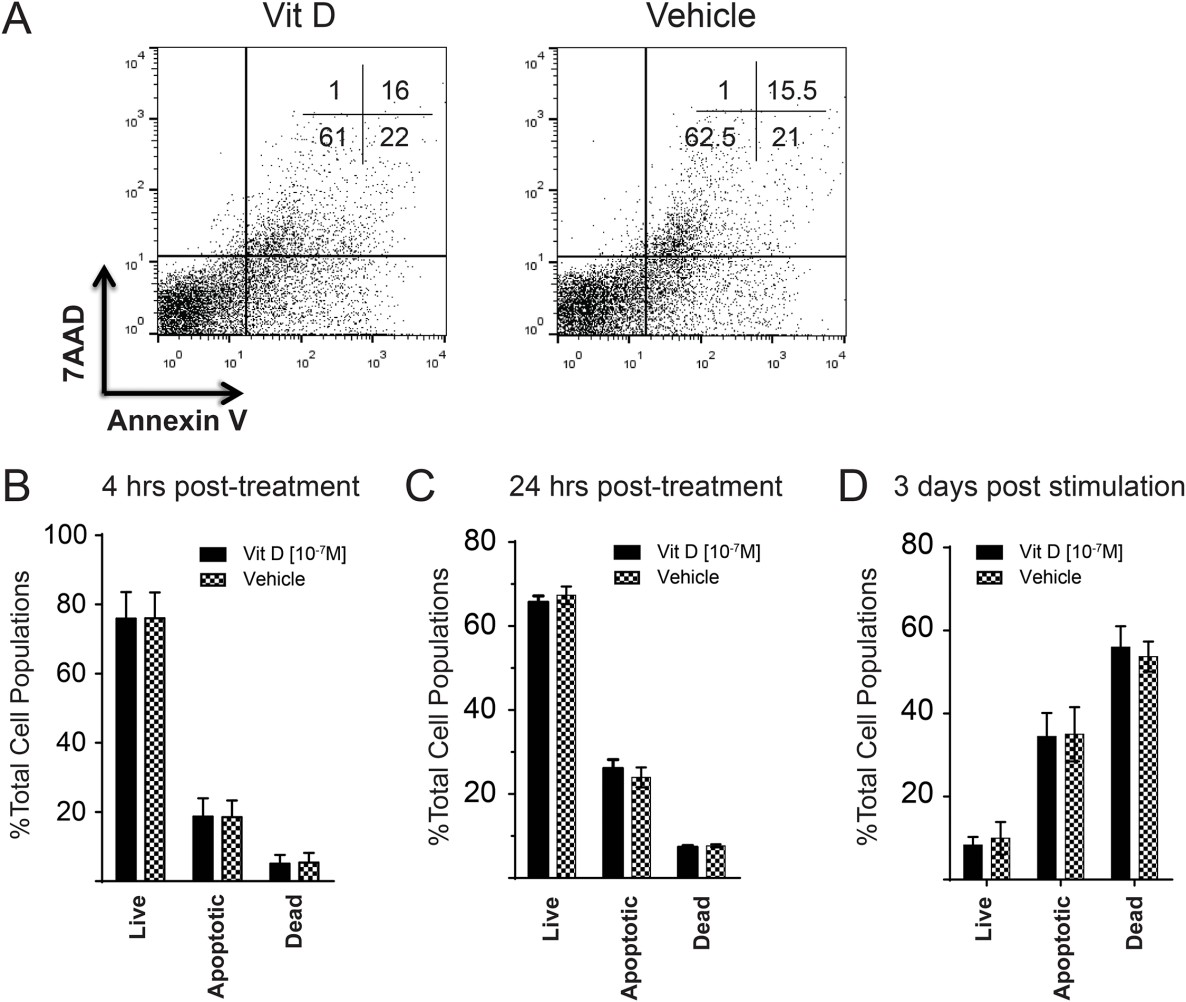
Vitamin D treatment does not induce cell death/apoptosis of chicken splenocytes. Chicken splenocytes were treated with Vitamin D (100nM) or vehicle (DMSO) for 4 hrs. The cells were stained with 7AAD (dead cell marker) and Annexin V (early apoptotic marker) and the data were analysed using flow cytometry. (**A**) Shows dot plots of the stained cells and the numbers in each quadrant represents percentage of cells. (**B**) The percentages (as mean ± SD) of live cells (Annexin V-7AAD- cells), apoptotic cells (Annexin V+7AAD- cells) and dead cells (Annexin V+7AAD+ cells) are shown from four independent experiments with three biological replicates in each experiment (12 biological replicates in total). (**C**) The data represent the percentage of live, apoptotic and dead cells 24hrs post Vitamin D (100 nM) or vehicle (DMSO) treatment. **(D)** The data represents the percentage of live, apoptotic, dead cells 3 days after Con A (10 μg/ml) stimulation.

To examine whether the Vitamin D induced inhibition of T cell proliferation and IFN-γ production is due to Vitamin D induced-cell death or apoptosis, mononuclear splenocytes were stained with 7AAD and Annexin V post Vitamin D (100 nM) pre-treatment. The percentages of live cells (7AAD^-^Annexin V^-^), apoptotic cells (7AAD^-^Annexin V^+^) and dead cells (7AAD^+^Annexin V^+^) were determined 4 hrs after the treatment using flow cytometry (Fig 2A). The data obtained from four independent experiments (with three biological repeats in each experiment) were combined. No significant difference was observed in the percentages of live, apoptotic or dead cells between Vitamin D (100 nM) and Vehicle (DMSO) pre-treatment cells (Fig 2B). To examine whether Vitamin D induces T cell apoptosis after a longer term treatment, the percentages of apoptotic and dead cells were analysed in the treated cells 24hrs (without stimulation; Fig 2C) or 72 hrs (after stimulation with Con A, 10 μg/ml; Fig 2D) post Vitamin D (100 nM, 10 nM) or vehicle treatment. No significant differences in the levels of apoptosis or cell death were observed at any time points, suggesting that Vitamin D does not induce T cells apoptosis. Taken together, the results demonstrate that the inhibitory effects of Vitamin D on T cell proliferation and IFN-γ production are not due to cell death or apoptosis.

### Vitamin D does not inhibit CD3^-^ or CD3^+^ T-cell degranulation

**Fig 3:**
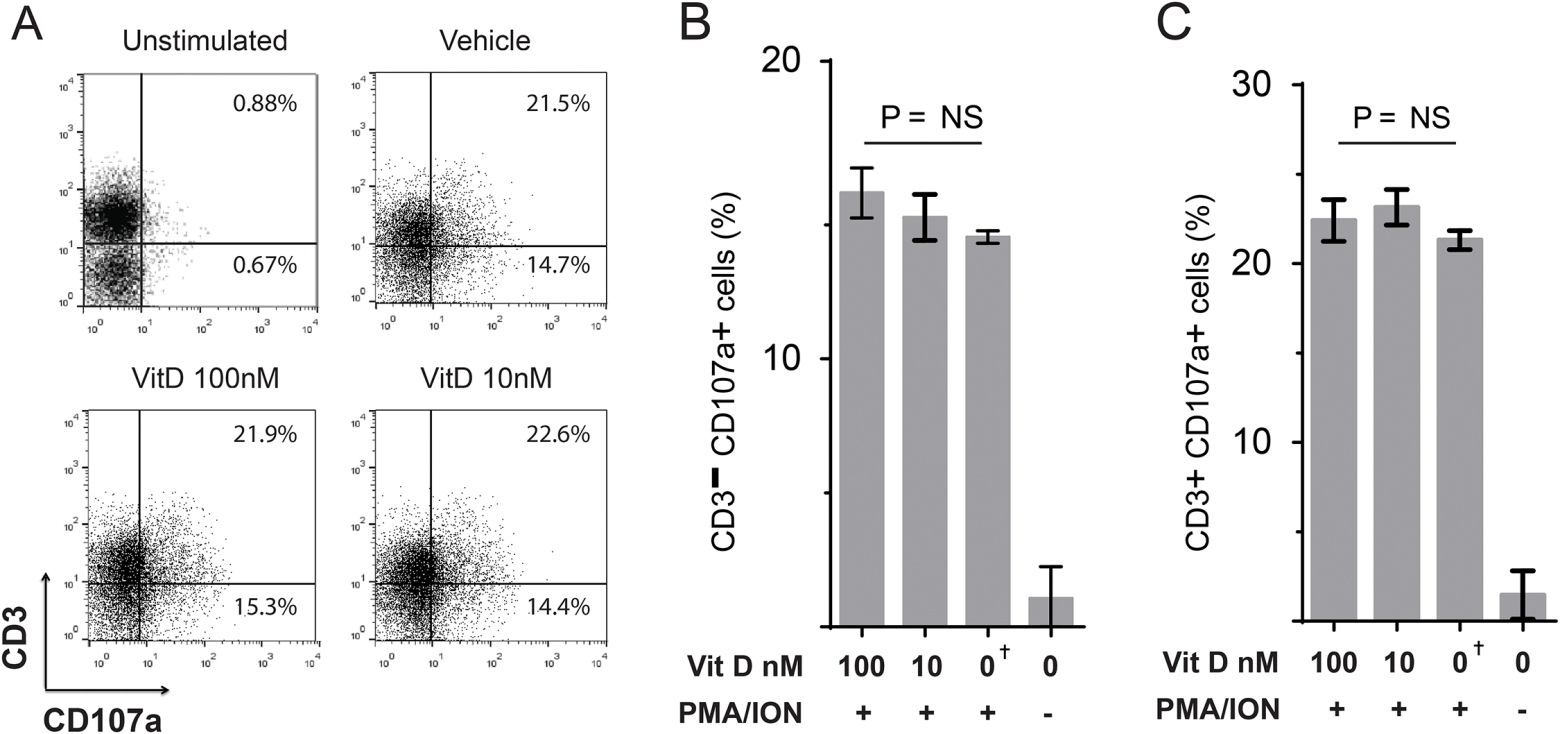
Degranulation of chicken T cells is not influenced by Vitamin D. (**A**) Representative flow cytometry dot plots of spleen mononuclear cells that were cultured in medium containing Vitamin D (100nM or 10 nM) or vehicle only (DMSO) for 4 hours and were stimulated with a T-cell stimulation cocktail of PMA and Ion for an additional 4 hrs. The expression of CD107a in CD3+ and CD3- cells was analysed using flow cytometry. Upper right quadrant and lower right quadrant shows the percentages of CD107a+ cells within CD3+ and CD3- T cells, respectively. The bar graphs represents the percentages of CD3-CD107a+. (**B**) and CD3+CD107a+ cells (**C**) in the treated cells. The results are shown as mean ± SD of the study population of CD107a expression (*P* = NS indicates no statistical significance). † (symbol) represents the cells treated with the Vehicle only (DMSO). Similar data were obtained in four independent experiments.

Translocation of lysosome-associated membrane protein 1 (LAMP1/CD107a) is a marker of degranulation, an important function of lymphocytes such as T cells and NK cells. We studied the effects of Vitamin D on the ability of CD3^-^ and CD3^+^ cells degranulation *in vitro*. Chicken mononuclear splenocytes were cultured in medium containing Vitamin D (100 nM or 10 nM) or vehicle only (DMSO) for 4 hrs, and were stimulated for an additional 4 hrs in medium with PMA (50 ng/ml) and ION (1μg/ml). The expression of CD107a was assessed on CD3^-^ cells and CD3^+^ T cells using flow cytometry (Fig 3A). The percentages of CD3^-^CD107a^+^ cells (Fig 3B) and CD3^+^CD107a^+^ T cells are shown (Fig 3C). The results demonstrate that Vitamin D (100 nM or 10 nM) has no significant effects on the cytotoxicity (degranulation) of CD3^-^ cells or CD3^+^ T cells *in vitro*. The results were confirmed in four independent experiments with three biological repeats in each experiment.

### Vitamin D limits ERK1/2 phosphorylation in unstimulated CD3^+^ T cells

**Fig 4:**
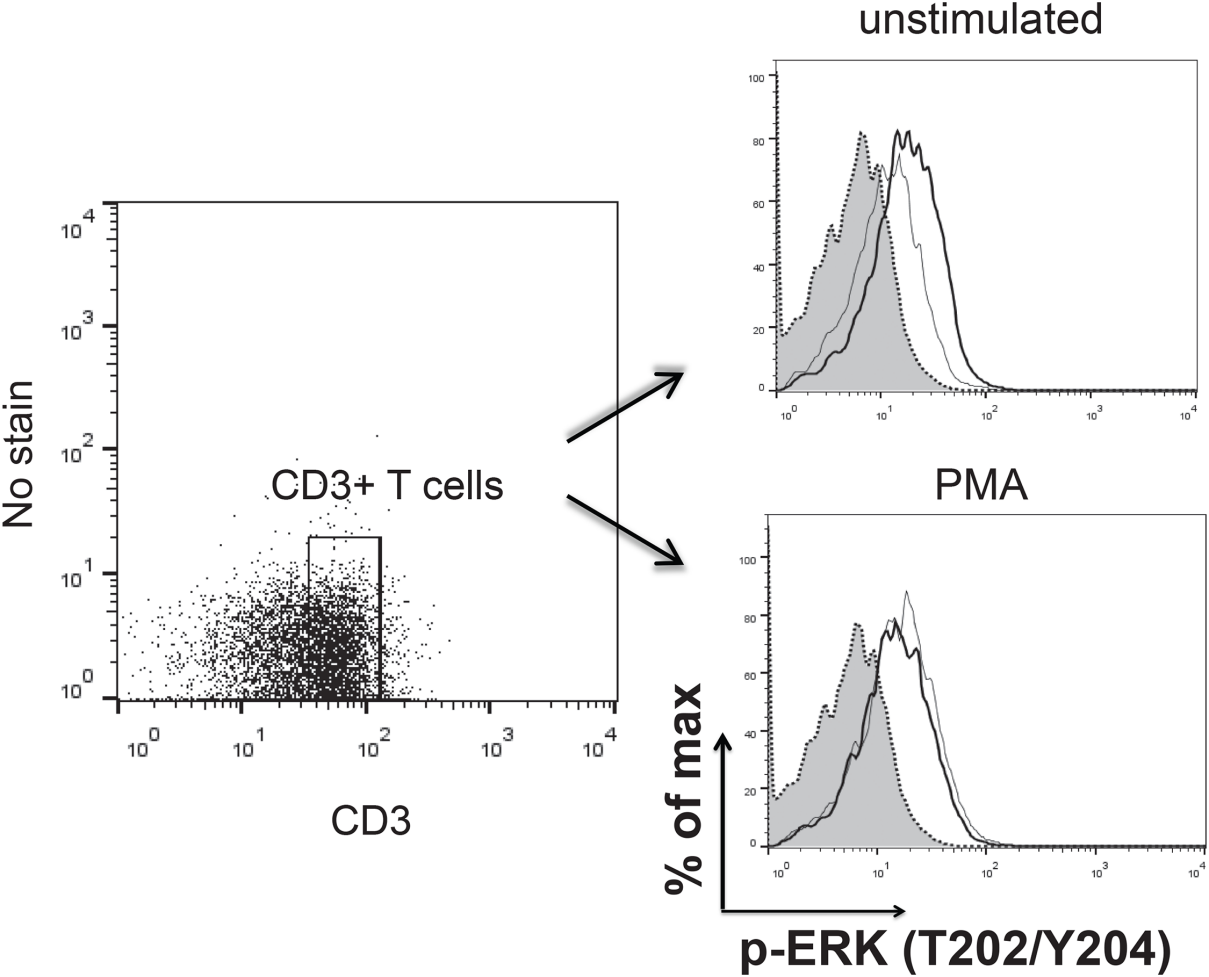
Vitamin D limits ERK1/2 phosphorylation in the resting chicken CD3+ T cells. Following incubation of splenocytes with Vitamin D or vehicle only for 4hrs, phosphorylation of ERK1/2 (T202/Y204) was evaluated in CD3+ T cells in the resting cells (unstimulated) or cells stimulated with PMA for 5 minutes using flow cytometry. The data represents flow cytometry analysis of ERK 1/2 phosphorylation in cells treated with vehicle (thick line), Vitamin D (thin line) or Isotype control (grey area) in the resting cells and PMA-stimulated T cells.

ERK1/2 signal pathway is an important regulator of T cell function. Here, we examined the effects of Vitamin D on phosphorylation of ERK1/2(T202/y204) in unstimulated and activated CD3^+^ T cells using Phosflow. Mononuclear splenocytes were cultured in medium containing Vitamin D (100 nM) or vehicle only for 4 hrs. PMA has been used in many studies for analysing human/ murine T cell signaling. In our hands, Con A cannot stimulate ERK1/2 phosphorylation and IFN-γ production by chicken T cells. Therefore, we decided to use PMA for analysing ERK 1/2 signalling. The phosphorylation of ERK1/2 (T202/y204) was analysed in the unstimulated cells and in the cells stimulated with PMA for 5 minutes. The results demonstrate that Vitamin D treatment reduces ERK1/2 phosphorylation in the unstimulated CD3^+^ T cells compared to the cells treated with vehicle only. However, there were no significant differences observed in the levels of ERK1/2 phosphorylation between Vitamin D and vehicle-treated cells after PMA stimulation (Fig 4). The results were confirmed in three independent experiments.

## Discussion

The immuno-modulatory, anti-inflammatory and cancer prevention properties of Vitamin D are attributed to its direct effects on immune system cells, including T lymphocytes in human and animal models [21, 37–41]. The results presented in this report demonstrate that the active Vitamin D metabolite modulates avian T lymphocytes effector functions similar to the results obtained from human and murine studies [14, 29, 42]. The functional heterogeneity of T lymphocytes is crucial for both limiting immuno-pathogenesis and promoting protective immune responses against pathogens. In this report, we demonstrate that Vitamin D reduces chicken T lymphocyte proliferation as well as the frequency of IFN-γ producing cells. The modulatory effects of Vitamin D were further confirmed based on down-regulation of ERK1/2 phosphorylation status that impacts subsequent downstream signal transduction pathways. Additionally, the results show that Vitamin D does not trigger programmed cell death by apoptosis, and its functional effects can only be attributed to the modulation of T lymphocyte effector function. In contrast to the inhibitory effects of Vitamin D on T cell proliferation and cytokine production, it did not have any significant effects on CD107a cell surface translocation, a marker for cytotoxic degranulation, confirming that Vitamin D does not induce general immuno-suppression of T cell functions. This notion was also confirmed with the results demonstrating that Vitamin D did not inhibit ERK1/2 phosphorylation in the activated T cells and only reduced base line ERK1/2 phosphorylation in the unstimulated cells.

T lymphocytes are the main adaptive immune cells and one of their main effector functions is their ability to proliferate in response to the stimuli or a specific antigen. Functional studies assessing Vitamin D effect on human immune system cells indicate a regulatory role as demonstrated by their ability to reduce the magnitude of T lymphocyte responses. Lemire *et al* [43] and Rigby *et al* [30] were among the first to demonstrate a dose dependent inhibition by Vitamin D on human PBMC proliferation after mitogen stimulation. Provvedini *et al* [8] provided the first evidence for the presence of a VDR in human immune system cells. However, little is known about the effect of Vitamin D on avian immune cells in health and disease and the consequent responses to the treatment. Our group and others have reported that Vitamin D may have both modulatory and stimulatory effects on chicken macrophages [25, 37, 44]. In this report, we present our data demonstrating that Vitamin D modulates T lymphocyte ability to proliferate in response to the stimuli. In human, the inhibition of T lymphocyte proliferation by Vitamin D has been linked with a reduction in IL-2 cytokine production [30]. Vitamin D interacts directly with its receptor (VDR), which has a VDR responsive element on the IL-2 gene, thereby repressing transcriptional activation [45, 46], which may explain in part its mechanism of action. Adding recombinant human IL-2 to the Vitamin D-treated T lymphocytes only partially restored T cell proliferative ability [30], suggesting that Vitamin D may exert its inhibitory effects in both IL-2 dependent and independent manner. A nuclear threshold mechanism for Vitamin D to promote VDR signalling is required to repress cytokine gene expression [2, 8]. On the other hand, supplementing Vitamin D restored human PBMC proliferative function in patients diagnosed as hypovitaminosis D [29]. Vitamin D also exerts immuno-regulatory effects on murine T cells. For example, it has been shown that pathogenic T lymphocytes are generated in VDR^-/-^ knockout mice due to overproduction of IL-2 resulting in excessive proliferation [47]. Moreover, in a multiple sclerosis (MS) model, T lymphocyte proliferation was inhibited via the addition of exogenous Vitamin D [38]. In addition, it has been demonstrated that Vitamin D reduces T lymphocyte effector function by generating dendritic cells that have lower ability to produce IL-12 [27, 42]. In this report, we show for the first time that Vitamin D is able to control the rate of avian T cell proliferation similar to what had been previously reported on human and murine T cells. It has previously been suggested that the effect of Vitamin D upon human T cells proliferation varies according to the strength of the stimulation and particularly affected by a lack of co-stimulation [48]. Based on the results obtained from Vitamin D-treated cells stimulated with different concentrations of the stimuli, we have demonstrated that Vitamin D does not increases the threshold of avian T cell stimulation, however further studies are required to determine the role of co-stimulatory molecules in activation of Vitamin D-treated avian T cells.

ERK1/2 is one of the foremost studied Mitogen-activated protein kinase (MAPK) within RAF-MEK-ERK signalling pathway in mammalian systems [49]. Integrated upstream signalling from TCR stimulation and cytokines activates the mechanism for ERK1/2 phosphorylation that transactivates transcriptional factors to regulate cellular proliferation at specific cell cycle checkpoint. In avian systems, specifically T cells, the complex MEK-ERK signalling pathway is scientifically compelling and hasn’t been explored to elucidate potential implication in immune responsiveness, pathogenesis and disease control. To our knowledge, this is the first report demonstrating that primary T lymphocytes treated with Vitamin D have lower ERK 1/2 (T202/Y204) phosphorylation levels at baseline in non-stimulated cells. ERK1/2 phosphorylation level was not affected in the stimulated T lymphocytes, suggesting that Vitamin D only reduces the baseline in the treated cells but these cells are fully responsive to the stimuli, confirming that Vitamin D may modulate avian immune responses but does not induce immuno-suppression or immune-unresponsiveness. There are two pathways which are potentially involved in the activation of ERK following TCR engagements or PMA activation ZAP-70 dependent and ZAP-70 independent pathways. Understanding the nature of intervening steps between TCR activation and effector function is incomplete. It is known that TCR engagement or stimulation of T cells with PMA leads to activation of the protein tyrosine kinase (PTK) ZAP-70, however it is still unclear how this process leads to the activation of ERK. Blocking ZAP-70 activation can inhibit T cell proliferation and effector function. Nevertheless, the activation of ZAP-70 negative T cells with PMA or TCR/CD3 stimulation can still induces ERK phosphorylation, suggesting that ERK activation is required but not enough for full T cell activation. These data may support our finding that Vitamin D inhibits T cell proliferation and effector function without inhibiting ERK activation in chicken T cells [50, 51]. Other stimuli might only activate one of the two activation pathways involved in ERK phosphorylation, which could be inhibited by Vitamin D treatment. This may explain why the activation of ERK pathway in human γδ T lymphocytes with isopentenyl pyrophosphate (a specific stimulant for γδ T lymphocytes) can be inhibited with Vitamin D treatment [52].

There is an increasing body of evidence reporting an anti-tumor role for Vitamin D by inducing apoptotic proteins in cancer cells from mice [39] or human tumours [40, 41]. These finding indicate that Vitamin D catabolism could differentially modulate tumour cell fate. However, there has been no report to date of Vitamin D-induced apoptosis in primary mammalian cells, and our data demonstrate that physiological levels of Vitamin D does not induces apoptosis of primary avian immune cells, including T cells. The physiological serum Vitamin D concentrations (25(OH)D3) in both healthy chickens [4, 53] and humans has been estimated to range between 60–100 nM [54–56], while the levels of serum 1,25(OH)_2_D3 are significantly lower. It has been shown that APCs and T cells are able to convert 25(OH)D3 to 1,25(OH)_2_D3 at physiologically relevant concentrations and respond to this in an autocrine fashion which can justify the supra-physiological concentration of 1,25(OH)_2_D3 used by several groups and including our study to analyse the effects of Vitamin D on T lymphocytes [55]. Further studies are required to establish whether avian tumour cells, similar to what is observed in human and murine cancer cells, are susceptible to Vitamin D induced apoptosis. These experiments are being planned in our laboratory with several tumour cell lines as well as primary tumour cells. Taken together, these results support our findings demonstrating that the reduction in frequency of IFN-γ producing cells and proliferation after Vitamin D treatment is not due to apoptosis of T cells, confirming that Vitamin D specifically inhibit the production of this cytokine from the activated T lymphocytes. IFN-γ is an antiviral cytokine and has an important role in the generation of anti-viral innate and adaptive immunity in both mammalian and avian systems. Lymphoid cells such as NK cells, γδ T cells, CD4+ T cells and CD8+ T cells are able to produce IFN-γ in response to the stimulation. The expression of IFN-γ significantly influences the initiation of anti-viral adaptive immunity, including the generation of effector Th1 cells [57]. In humans, Vitamin D has been used successfully in the prevention and control of viral infections [20, 21, 58]. Reichel *et al* [59] demonstrated the inhibitory effects of Vitamin D on human T cells ability to produce IFN-γ in a dose dependent manner [60]. In clinical settings, a reduction in IFN-γ production may reduce immunopathology observed in autoimmune and acute or chronic inflammatory diseases induced by infectious agents. Vitamin D provides a homeostatic framework required for generation of a protective immune response against infections without causing immunopathology. Adaptive immune system cells such as T and B cells have been shown to express VDR and constitutively transcribe the 1αhydroxylase enzyme required for producing the active Vitamin D molecule [8, 11, 35, 45]. Therefore Vitamin D can directly modulate T cell effector function. However, Vitamin D may also indirectly affect avian T cell function via generation of immuno-modulatory antigen presenting cells [44]. In-vitro exposure to Vitamin D can sustain suppression of T lymphocyte transcriptional activation and consequent production of IFN-γ mRNA in long-term culture systems [60], which could be of particular importance in over-activated T lymphocytes. These findings may have significant importance in avian systems and needs to be further explored. Restoring T lymphocyte functional abilities could be key in defence against pathogens as previously outlines in human PBMC proliferation [29].

Additionally, we evaluated cell surface co-mobilization of lytic granule membrane protein CD107a to study activation of cytotoxic T lymphocytes (CTLs) when treated with Vitamin D. CTLs play an import role in defence against viral infections as well as cancer. Activation of CTLs leads to degranulation and secretion of lytic granules containing perforin and granzymes. Recently, CD107a cell surface mobilization has been described as a marker for cytotoxic T cell degranulation and shown to be strongly up regulated in activated T cells [61]. Human natural killer (NK) cells cytotoxic function has been characterized based on a positive association between stimulatory cytokines such as IL-2 and IFN-γ and up-regulation of CD107a to the cell surface [62]. Experimental evidence suggests an increase in cell surface expression of CD107a in PBMCs of patients with autoimmune diseases. In avian system, CD107a is also a marker for cytotoxic activity of T cells [63] and NK cells [64], and has been suggested to be important in the control of viral infections such as Infectious Bronchitis Virus (IBV) by reducing viral load. Here, we demonstrate that physiological levels of Vitamin D do not alter cytotoxic abilities of lymphocytes, and the levels of degranulation in CD3^+^ T-cells (including CD4^+^ and CD8^+^ T cells) or CD3^-^ lymphocytes (including NK cells) are not affected by Vitamin D treatment. It seems that IFN-γ production and degranulation are differentially regulated in avian T cells and non-T cells. Taken together, our results demonstrate that Vitamin D preferentially down-regulate the production of pro-inflammatory cytokines such as IFN-γ but does not inhibit their cytotoxic abilities, which is required for control of the infected cells or transformed cells. Degranulation and cytokine production can be differentially detected in different maturation stages of murine and human lymphocytes. For example, CD11b^low^CD27^high^ murine NK cells preferentially degranulate while CD11b^high^CD27^low^ NK cells display reduced degranulation but with maintained IFN-γ production [65, 66]. Preferential function of different maturation stages of human NK cells has also been reported, in which CD56^bright^CD16^-^ NK cells (immature NK cells) are abundant cytokine producers, while they are less effective in degranulation and cytotoxicity [67]. It is possible, but not proven, that Vitamin D exerts its preferential effects on CD3^-^ lymphocyte function by modulating the maturation stages of these cells. The effects of Vitamin D on maturation stages of immune system cells was evident from our previous results demonstrating that Vitamin D inhibits up-regulation of maturation markers such as CD86, CD80 and MHC class II on avian macrophages, while increasing their abilities to produce nitric oxide [44]. The activation stage of antigen specific CD8^+^ T cells can also influence the preferential functional abilities of CTLs. Fully differentiated CTLs can undergo degranulation without producing IFN-γ, while many effector memory CD8^+^ T cells produced IFN-γ but are not able to degranulate in response to stimuli [68]. There is no information on the effects of Vitamin D on the activation stages of murine or human T cells. However, the results presented in this report indicate that Vitamin D-treated avian T lymphocytes may have functional abilities as fully differentiated T cells, with reduced frequency of IFN-γ secretion, but retain their ability to undergo degranulation.

Respiratory infections are the major problems in poultry farms, with many possible causes including viral, bacterial and fungal. In humans, the peak incidence of respiratory tract infection coincides with the time of the year when there is insufficient UV-B light to produce Vitamin D resulting in low serum Vitamin D levels in the population [69, 70]. Based on these aforementioned epidemiological assessments, indoor housed chickens may be at greater risk for respiratory infection and this might be explained by many mechanisms including variation in the levels of Vitamin D [4]. It is now believed that the effects of Vitamin D on innate and adaptive immunity may play an important role in controlling seasonal respiratory infections [71].

In conclusion, we demonstrated that Vitamin D can modulate the function of avian T lymphocytes by reducing T cell proliferation, cytokine production and a reduction in phosphorylation of non-stimulated T lymphocytes. However, Vitamin D treatment did not alter the ability of T lymphocyte to undergo degranulation and did not suppress ERK1/2 phosphorylation in response to the stimuli. This may be due to differential effects of vitamin D on the functional abilities of T lymphocytes, which can inhibit immunopathology that leads to exhaustion of T lymphocytes without inducing general immunosuppression.
